# 1-[2,2-Bis(phenyl­sulfon­yl)ethen­yl]-4-meth­oxy­benzene

**DOI:** 10.1107/S1600536812001961

**Published:** 2012-01-21

**Authors:** Haruyasu Asahara, Peter Mayer, Herbert Mayr

**Affiliations:** a Ludwig-Maximilians-Universität, Department of Chemistry, Butenandtstrasse 5–13, 81377 München, Germany

## Abstract

In the title compound, C_21_H_18_O_5_S_2_, the two sulfur-bound phenyl rings lie on opposite sides of the meth­oxy­phenyl group, making dihedral angles of 77.58 (8) and 87.45 (8)°with it. The dihedral angle between the sulfur-bound phenyl rings is 57.31 (8)°. In the crystal, π–π stacking is observed between the two sulfur-bound phenyl rings, with a centroid–centroid distance of 3.878 (1) Å and a dihedral angle of 7.58 (8)°. The mol­ecules are linked by weak C—H⋯O and C—H⋯π contacts.

## Related literature

For background to bis­sulfonyl ethyl­enes and their synthesis, see: Simpkins (1993 ▶); Najera & Yus (1999 ▶); Prilezhaeva (2000 ▶); Nielsen *et al.* (2010 ▶), Zhu & Lu (2009 ▶), Alba *et al.* (2010 ▶), Sulzer-Moss *et al.* (2009 ▶). For a related structure, see: De Lucchi *et al.* (1985 ▶).
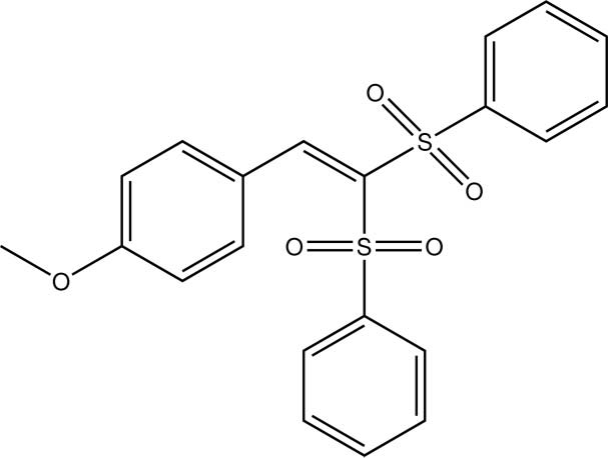


## Experimental

### 

#### Crystal data

C_21_H_18_O_5_S_2_
*M* *_r_* = 414.50
Monoclinic, 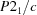

*a* = 7.8291 (1) Å
*b* = 21.6666 (4) Å
*c* = 12.0332 (2) Å
β = 107.8449 (10)°
*V* = 1942.99 (5) Å^3^
*Z* = 4
Mo *K*α radiation
μ = 0.31 mm^−1^
*T* = 173 K
0.33 × 0.26 × 0.21 mm

#### Data collection

Nonius KappaCCD diffractometer
15675 measured reflections
4445 independent reflections
3908 reflections with *I* > 2σ(*I*)
*R*_int_ = 0.026

#### Refinement

*R*[*F* ^2^ > 2σ(*F* ^2^)] = 0.033
*wR*(*F* ^2^) = 0.084
*S* = 1.08
4445 reflections
254 parameters
H-atom parameters constrained
Δρ_max_ = 0.33 e Å^−3^
Δρ_min_ = −0.38 e Å^−3^

### 

Data collection: *COLLECT* (Hooft, 2004 ▶); cell refinement: *SCALEPACK* (Otwinowski & Minor, 1997 ▶); data reduction: *DENZO* (Otwinowski & Minor, 1997 ▶) and *SCALEPACK*; program(s) used to solve structure: *SIR97* (Altomare *et al.*, 1999 ▶); program(s) used to refine structure: *SHELXL97* (Sheldrick, 2008 ▶); molecular graphics: *ORTEP-3* (Farrugia, 1997 ▶) and *Mercury* (Macrae *et al.*, 2006 ▶); software used to prepare material for publication: *PLATON* (Spek, 2009 ▶).

## Supplementary Material

Crystal structure: contains datablock(s) I, global. DOI: 10.1107/S1600536812001961/zj2048sup1.cif

Structure factors: contains datablock(s) I. DOI: 10.1107/S1600536812001961/zj2048Isup2.hkl

Supplementary material file. DOI: 10.1107/S1600536812001961/zj2048Isup3.cml

Additional supplementary materials:  crystallographic information; 3D view; checkCIF report

## Figures and Tables

**Table 1:** Hydrogen-bond geometry (Å, °) *Cg* is the centroid of the C16–C21 ring.

*D*—H⋯*A*	*D*—H	H⋯*A*	*D*⋯*A*	*D*—H⋯*A*
C8—H8⋯*Cg*1^i^	0.95	2.56	3.4835 (17)	164
C14—H14⋯O1^ii^	0.95	2.51	3.229 (2)	133
C21—H21⋯O3^i^	0.95	2.50	3.2695 (19)	138
C20—H20⋯O4^iii^	0.95	2.59	3.453 (2)	151

Symmetry codes: (i) 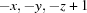; (ii) 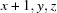; (iii) 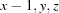.

**Fig. 1.**
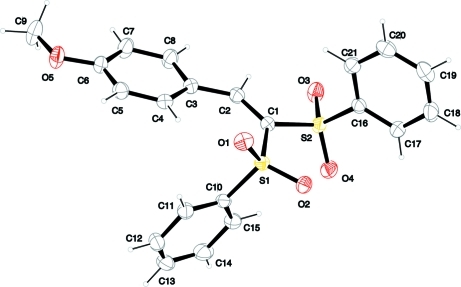
The molecular structure of the title compound, with atom labels and anisotropic displacement ellipsoids (drawn at 50% probability level) for non-H atoms.

**Fig. 2.**
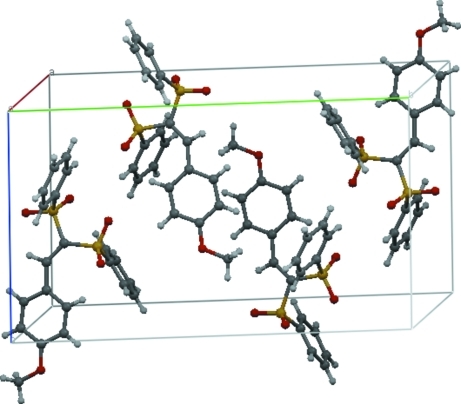
The packing of the title compound.

## supplementary crystallographic information

### Comment

Bissulfonyl ethylenes are important reagents in synthetic organic chemistry, because they are active Michael acceptors [Simpkins (1993), Najera *et al.* (1999), Prilezhaeva (2000)]. Recently, organocatalytic Michael additions of bissulfonyl ethylene have also been reported [Nielsen *et al.* (2010), Zhu *et al.* (2009), Alba *et al.* (2010)]. During our studies on the electrophilic reactivity of bissulfonyl ethylenes, we discussed structure-reactivity relationships.

In the title compound, the C1—C2 double bond deviates only slighthly from coplanarity with the phenyl ring of the methoxyphenyl group (plane-bond angle 10.22 (10)°). The double bonds S1—O2 and S2—O3 are coplanar with the C1—C2 double bond as is indicated by the torsion angles O2—S1—C1—C2 (-178.13 (13)°) and O3—S2—C1—C2 (1.12 (13)°). The sulfur-bound phenyl rings lie to opposite sides of the methoxyphenyl group with dihedral angles of 77.58 (8)° and 87.45 (8)°. The ring bound to S1 is almost coplanar with the S1—O1 double bond (plane-bond angle 7.79 (8)°), the ring bound to S2 is nearly coplanar with the S2—O4 double bond (plane-bond angle 9.12 (7)°). The molecular structure of the title compound is shown in Figure 1.

The packing of the title compound is shown in Figure 2. π-π-stacking is observed between the two sulfur-bound phenyl rings with a centroid-centroid distance of 3.878 (1) Å and a dihedral angle of 7.58 (8)°. A C–H···π contact is established between the phenyl ring bound to S2 and the C8–H8 moiety. The distance of H8 to the centre of gravity of the phenyl ring is 2.56 Å, the angle around H8 is 164°. Furthermore weak C–H···O contacts with sulfur-bound oxygen atoms as acceptors are observed.

### Experimental

The title compound has been obtained by following modified method of Alexakis [Sulzer-Moss *et al.* (2009)]. A mixture of *p*-anisaldehyde (15.0 g, 110 mmol, 7.4 equiv.), bis(phenylsulfonyl)methane (4.4 g, 14.8 mmol, 1.0 equiv.), diethylammonium chloride (32.1 mmol, 2.1 equiv.) and potassium fluoride (2.5 mmol, 0.17 equiv.) in dry toluene (150 ml) was stirred and refluxed under a Dean Stark water separator for 24 h. After cooling, the solvent was evaporated and residue was partitioned between water (50 ml) and CH_2_Cl_2_ (150 ml). The organic phase was separated and the aqueous phase was extracted with CH_2_Cl_2_ (three times 25 ml). The combined organic layer was dried over Na_2_SO_4_, filtered and concentrated under reduced pressure. The crude mixture was purified by flash column chromatography on silica gel (pentane/ethyl acetate: from 95/5 to 80/20), followed by recrystallization from pentane/chloroform. mp 123.0–123.9 °C (yield 4.9 g, 11.8 mmol, 79.9%).

### Refinement

C-bound H atoms were positioned geometrically (C—H = 0.98 Å for aliphatic, 0.95 Å for aromatic H) and treated as riding on their parent atoms [*U*_iso_(H) = 1.2*U*_eq_(C, aromatic), *U*_iso_(H) = 1.5*U*_eq_(C, aliphatic)].

### Crystal data

**Table:**

C_21_H_18_O_5_S_2_	*F*(000) = 864
*M**_r_* = 414.50	*D*_x_ = 1.417 (1) Mg m^−^^3^
Monoclinic, *P*2_1_/*c*	Mo *K*α radiation, λ = 0.71073 Å
Hall symbol: -P 2ybc	Cell parameters from 7909 reflections
*a* = 7.8291 (1) Å	θ = 3.1–27.5°
*b* = 21.6666 (4) Å	µ = 0.31 mm^−^^1^
*c* = 12.0332 (2) Å	*T* = 173 K
β = 107.8449 (10)°	Block, yellow
*V* = 1942.99 (5) Å^3^	0.33 × 0.26 × 0.21 mm
*Z* = 4	

### Data collection

**Table:**

Nonius KappaCCD diffractometer	3908 reflections with *I* > 2σ(*I*)
Radiation source: rotating anode	*R*_int_ = 0.026
MONTEL, graded multilayered X-ray optics	θ_max_ = 27.5°, θ_min_ = 3.3°
CCD; rotation images; thick slices scans	*h* = −10→10
15675 measured reflections	*k* = −27→28
4445 independent reflections	*l* = −15→15

### Refinement

**Table:**

Refinement on *F*^2^	Primary atom site location: structure-invariant direct methods
Least-squares matrix: full	Secondary atom site location: difference Fourier map
*R*[*F*^2^ > 2σ(*F*^2^)] = 0.033	Hydrogen site location: inferred from neighbouring sites
*wR*(*F*^2^) = 0.084	H-atom parameters constrained
*S* = 1.08	*w* = 1/[σ^2^(*F*_o_^2^) + (0.031*P*)^2^ + 1.0861*P*] where *P* = (*F*_o_^2^ + 2*F*_c_^2^)/3
4445 reflections	(Δ/σ)_max_ < 0.001
254 parameters	Δρ_max_ = 0.33 e Å^−^^3^
0 restraints	Δρ_min_ = −0.38 e Å^−^^3^

### Special details

**Table:**

Geometry. All e.s.d.'s (except the e.s.d. in the dihedral angle between two l.s. planes) are estimated using the full covariance matrix. The cell e.s.d.'s are taken into account individually in the estimation of e.s.d.'s in distances, angles and torsion angles; correlations between e.s.d.'s in cell parameters are only used when they are defined by crystal symmetry. An approximate (isotropic) treatment of cell e.s.d.'s is used for estimating e.s.d.'s involving l.s. planes.
Refinement. Refinement of *F*^2^ against ALL reflections. The weighted *R*-factor *wR* and goodness of fit *S* are based on *F*^2^, conventional *R*-factors *R* are based on *F*, with *F* set to zero for negative *F*^2^. The threshold expression of *F*^2^ > 2*σ*(*F*^2^) is used only for calculating *R*-factors(gt) *etc*. and is not relevant to the choice of reflections for refinement. *R*-factors based on *F*^2^ are statistically about twice as large as those based on *F*, and *R*-factors based on all data will be even larger.

### Fractional atomic coordinates and isotropic or equivalent isotropic displacement parameters (Å^2^)

**Table:**

	*x*	*y*	*z*	*U*_iso_*/*U*_eq_	
S1	0.20814 (5)	0.197024 (16)	0.62810 (3)	0.02090 (9)	
S2	0.18614 (5)	0.089334 (17)	0.46455 (3)	0.02332 (10)	
O1	0.07863 (14)	0.21777 (5)	0.68234 (10)	0.0292 (2)	
O2	0.20435 (14)	0.22512 (5)	0.51907 (9)	0.0278 (2)	
O3	0.17420 (16)	0.02302 (5)	0.46677 (9)	0.0313 (3)	
O4	0.33662 (14)	0.11588 (5)	0.43740 (9)	0.0305 (3)	
O5	0.25877 (18)	0.04667 (6)	1.16063 (9)	0.0365 (3)	
C1	0.18455 (19)	0.11624 (7)	0.60485 (12)	0.0214 (3)	
C2	0.1764 (2)	0.07226 (7)	0.68278 (13)	0.0245 (3)	
H2	0.1564	0.0325	0.6481	0.029*	
C3	0.1902 (2)	0.07015 (7)	0.80624 (12)	0.0242 (3)	
C4	0.18860 (19)	0.12011 (7)	0.88083 (13)	0.0243 (3)	
H4	0.1734	0.1609	0.8506	0.029*	
C5	0.2090 (2)	0.11019 (7)	0.99738 (13)	0.0276 (3)	
H5	0.2058	0.1443	1.0464	0.033*	
C6	0.2341 (2)	0.05075 (7)	1.04444 (13)	0.0274 (3)	
C7	0.2320 (2)	0.00049 (8)	0.97225 (14)	0.0338 (4)	
H7	0.2464	−0.0403	1.0027	0.041*	
C8	0.2085 (2)	0.01103 (7)	0.85489 (14)	0.0322 (4)	
H8	0.2045	−0.0234	0.8054	0.039*	
C9	0.2945 (3)	−0.01316 (9)	1.21382 (15)	0.0435 (4)	
H9A	0.1915	−0.0403	1.1800	0.065*	
H9B	0.3150	−0.0094	1.2981	0.065*	
H9C	0.4014	−0.0307	1.1998	0.065*	
C10	0.42503 (19)	0.20496 (7)	0.72786 (13)	0.0227 (3)	
C11	0.4505 (2)	0.23657 (7)	0.83213 (13)	0.0284 (3)	
H11	0.3517	0.2532	0.8523	0.034*	
C12	0.6252 (2)	0.24312 (8)	0.90616 (14)	0.0365 (4)	
H12	0.6463	0.2645	0.9780	0.044*	
C13	0.7677 (2)	0.21894 (9)	0.87638 (15)	0.0394 (4)	
H13	0.8863	0.2241	0.9275	0.047*	
C14	0.7396 (2)	0.18709 (9)	0.77248 (16)	0.0368 (4)	
H14	0.8387	0.1701	0.7531	0.044*	
C15	0.5677 (2)	0.17990 (8)	0.69699 (14)	0.0283 (3)	
H15	0.5474	0.1583	0.6254	0.034*	
C16	−0.0154 (2)	0.11649 (7)	0.36510 (12)	0.0231 (3)	
C17	−0.0075 (2)	0.14543 (7)	0.26401 (13)	0.0298 (3)	
H17	0.1047	0.1546	0.2526	0.036*	
C18	−0.1677 (2)	0.16064 (8)	0.17987 (14)	0.0361 (4)	
H18	−0.1656	0.1801	0.1096	0.043*	
C19	−0.3298 (2)	0.14774 (8)	0.19744 (15)	0.0355 (4)	
H19	−0.4383	0.1582	0.1390	0.043*	
C20	−0.3363 (2)	0.11958 (8)	0.29952 (15)	0.0323 (4)	
H20	−0.4487	0.1113	0.3112	0.039*	
C21	−0.1780 (2)	0.10357 (7)	0.38461 (14)	0.0270 (3)	
H21	−0.1806	0.0842	0.4549	0.032*	

### Atomic displacement parameters (Å^2^)

**Table:**

	*U*^11^	*U*^22^	*U*^33^	*U*^12^	*U*^13^	*U*^23^
S1	0.01895 (17)	0.02019 (17)	0.02280 (18)	0.00109 (13)	0.00526 (13)	0.00185 (13)
S2	0.02724 (19)	0.02359 (19)	0.01953 (17)	0.00020 (14)	0.00776 (14)	0.00026 (13)
O1	0.0256 (5)	0.0272 (6)	0.0373 (6)	0.0061 (4)	0.0134 (5)	0.0010 (5)
O2	0.0302 (6)	0.0260 (6)	0.0252 (5)	0.0007 (4)	0.0054 (4)	0.0073 (4)
O3	0.0453 (7)	0.0237 (6)	0.0250 (5)	0.0030 (5)	0.0112 (5)	−0.0005 (4)
O4	0.0279 (6)	0.0390 (7)	0.0275 (6)	−0.0007 (5)	0.0125 (5)	0.0005 (5)
O5	0.0530 (7)	0.0367 (7)	0.0210 (5)	0.0024 (6)	0.0133 (5)	0.0005 (5)
C1	0.0213 (7)	0.0219 (7)	0.0204 (6)	−0.0013 (5)	0.0056 (5)	−0.0011 (5)
C2	0.0267 (7)	0.0229 (7)	0.0231 (7)	−0.0024 (6)	0.0062 (6)	−0.0017 (6)
C3	0.0261 (7)	0.0244 (7)	0.0218 (7)	−0.0024 (6)	0.0069 (6)	−0.0006 (6)
C4	0.0240 (7)	0.0232 (7)	0.0261 (7)	−0.0021 (6)	0.0082 (6)	0.0002 (6)
C5	0.0305 (8)	0.0274 (8)	0.0260 (7)	−0.0012 (6)	0.0102 (6)	−0.0053 (6)
C6	0.0297 (8)	0.0334 (8)	0.0198 (7)	−0.0022 (6)	0.0085 (6)	−0.0004 (6)
C7	0.0511 (10)	0.0261 (8)	0.0255 (8)	0.0004 (7)	0.0139 (7)	0.0027 (6)
C8	0.0503 (10)	0.0233 (8)	0.0247 (8)	−0.0026 (7)	0.0138 (7)	−0.0033 (6)
C9	0.0634 (12)	0.0432 (11)	0.0268 (8)	0.0097 (9)	0.0180 (8)	0.0097 (7)
C10	0.0209 (7)	0.0218 (7)	0.0241 (7)	−0.0031 (5)	0.0046 (5)	0.0024 (5)
C11	0.0342 (8)	0.0242 (8)	0.0261 (7)	−0.0037 (6)	0.0080 (6)	0.0004 (6)
C12	0.0448 (10)	0.0330 (9)	0.0248 (8)	−0.0110 (8)	0.0005 (7)	0.0012 (7)
C13	0.0305 (9)	0.0432 (10)	0.0349 (9)	−0.0134 (7)	−0.0039 (7)	0.0104 (8)
C14	0.0220 (8)	0.0451 (10)	0.0419 (10)	−0.0015 (7)	0.0077 (7)	0.0094 (8)
C15	0.0234 (7)	0.0325 (8)	0.0289 (8)	−0.0020 (6)	0.0079 (6)	0.0013 (6)
C16	0.0272 (7)	0.0200 (7)	0.0202 (7)	−0.0025 (6)	0.0047 (6)	−0.0018 (5)
C17	0.0345 (8)	0.0296 (8)	0.0243 (7)	−0.0056 (7)	0.0076 (6)	0.0028 (6)
C18	0.0448 (10)	0.0343 (9)	0.0237 (8)	−0.0024 (7)	0.0022 (7)	0.0053 (6)
C19	0.0344 (9)	0.0308 (9)	0.0324 (8)	0.0027 (7)	−0.0030 (7)	−0.0033 (7)
C20	0.0280 (8)	0.0289 (8)	0.0386 (9)	−0.0007 (6)	0.0082 (7)	−0.0083 (7)
C21	0.0313 (8)	0.0233 (7)	0.0273 (7)	−0.0020 (6)	0.0105 (6)	−0.0024 (6)

### Geometric parameters (Å, °)

**Table:**

S1—O1	1.4359 (11)	C9—H9B	0.9800
S1—O2	1.4383 (11)	C9—H9C	0.9800
S1—C10	1.7620 (15)	C10—C11	1.389 (2)
S1—C1	1.7731 (15)	C10—C15	1.391 (2)
S2—O4	1.4357 (11)	C11—C12	1.392 (2)
S2—O3	1.4405 (12)	C11—H11	0.9500
S2—C16	1.7638 (15)	C12—C13	1.375 (3)
S2—C1	1.7897 (14)	C12—H12	0.9500
O5—C6	1.3544 (18)	C13—C14	1.385 (3)
O5—C9	1.435 (2)	C13—H13	0.9500
C1—C2	1.352 (2)	C14—C15	1.382 (2)
C2—C3	1.457 (2)	C14—H14	0.9500
C2—H2	0.9500	C15—H15	0.9500
C3—C8	1.397 (2)	C16—C17	1.387 (2)
C3—C4	1.409 (2)	C16—C21	1.392 (2)
C4—C5	1.379 (2)	C17—C18	1.389 (2)
C4—H4	0.9500	C17—H17	0.9500
C5—C6	1.396 (2)	C18—C19	1.377 (3)
C5—H5	0.9500	C18—H18	0.9500
C6—C7	1.390 (2)	C19—C20	1.386 (2)
C7—C8	1.386 (2)	C19—H19	0.9500
C7—H7	0.9500	C20—C21	1.388 (2)
C8—H8	0.9500	C20—H20	0.9500
C9—H9A	0.9800	C21—H21	0.9500
			
O1—S1—O2	117.52 (7)	O5—C9—H9C	109.5
O1—S1—C10	109.09 (7)	H9A—C9—H9C	109.5
O2—S1—C10	109.05 (7)	H9B—C9—H9C	109.5
O1—S1—C1	109.08 (7)	C11—C10—C15	121.94 (14)
O2—S1—C1	107.57 (7)	C11—C10—S1	120.33 (12)
C10—S1—C1	103.64 (7)	C15—C10—S1	117.71 (11)
O4—S2—O3	117.79 (7)	C10—C11—C12	117.97 (15)
O4—S2—C16	109.75 (7)	C10—C11—H11	121.0
O3—S2—C16	107.20 (7)	C12—C11—H11	121.0
O4—S2—C1	109.11 (7)	C13—C12—C11	120.72 (16)
O3—S2—C1	106.75 (7)	C13—C12—H12	119.6
C16—S2—C1	105.54 (7)	C11—C12—H12	119.6
C6—O5—C9	117.78 (13)	C12—C13—C14	120.50 (15)
C2—C1—S1	127.66 (11)	C12—C13—H13	119.8
C2—C1—S2	116.11 (11)	C14—C13—H13	119.8
S1—C1—S2	116.10 (8)	C15—C14—C13	120.17 (17)
C1—C2—C3	136.46 (14)	C15—C14—H14	119.9
C1—C2—H2	111.8	C13—C14—H14	119.9
C3—C2—H2	111.8	C14—C15—C10	118.70 (15)
C8—C3—C4	117.20 (14)	C14—C15—H15	120.7
C8—C3—C2	114.97 (14)	C10—C15—H15	120.7
C4—C3—C2	127.82 (14)	C17—C16—C21	121.88 (14)
C5—C4—C3	120.49 (14)	C17—C16—S2	118.33 (12)
C5—C4—H4	119.8	C21—C16—S2	119.53 (11)
C3—C4—H4	119.8	C16—C17—C18	118.24 (15)
C4—C5—C6	120.95 (14)	C16—C17—H17	120.9
C4—C5—H5	119.5	C18—C17—H17	120.9
C6—C5—H5	119.5	C19—C18—C17	120.58 (16)
O5—C6—C7	124.39 (15)	C19—C18—H18	119.7
O5—C6—C5	115.88 (14)	C17—C18—H18	119.7
C7—C6—C5	119.73 (14)	C18—C19—C20	120.75 (15)
C8—C7—C6	118.70 (15)	C18—C19—H19	119.6
C8—C7—H7	120.7	C20—C19—H19	119.6
C6—C7—H7	120.7	C19—C20—C21	119.75 (16)
C7—C8—C3	122.86 (15)	C19—C20—H20	120.1
C7—C8—H8	118.6	C21—C20—H20	120.1
C3—C8—H8	118.6	C20—C21—C16	118.78 (15)
O5—C9—H9A	109.5	C20—C21—H21	120.6
O5—C9—H9B	109.5	C16—C21—H21	120.6
H9A—C9—H9B	109.5		
			
O1—S1—C1—C2	−49.65 (15)	O1—S1—C10—C11	−9.58 (14)
O2—S1—C1—C2	−178.13 (13)	O2—S1—C10—C11	119.97 (12)
C10—S1—C1—C2	66.46 (15)	C1—S1—C10—C11	−125.68 (12)
O1—S1—C1—S2	134.70 (8)	O1—S1—C10—C15	171.88 (12)
O2—S1—C1—S2	6.22 (10)	O2—S1—C10—C15	−58.57 (13)
C10—S1—C1—S2	−109.19 (9)	C1—S1—C10—C15	55.78 (13)
O4—S2—C1—C2	−127.17 (12)	C15—C10—C11—C12	0.4 (2)
O3—S2—C1—C2	1.12 (13)	S1—C10—C11—C12	−178.05 (12)
C16—S2—C1—C2	114.95 (12)	C10—C11—C12—C13	0.0 (2)
O4—S2—C1—S1	49.00 (10)	C11—C12—C13—C14	−0.6 (3)
O3—S2—C1—S1	177.29 (8)	C12—C13—C14—C15	0.7 (3)
C16—S2—C1—S1	−68.88 (9)	C13—C14—C15—C10	−0.3 (3)
S1—C1—C2—C3	−3.5 (3)	C11—C10—C15—C14	−0.3 (2)
S2—C1—C2—C3	172.18 (15)	S1—C10—C15—C14	178.21 (12)
C1—C2—C3—C8	−167.66 (18)	O4—S2—C16—C17	14.62 (14)
C1—C2—C3—C4	11.9 (3)	O3—S2—C16—C17	−114.41 (12)
C8—C3—C4—C5	1.6 (2)	C1—S2—C16—C17	132.07 (12)
C2—C3—C4—C5	−177.91 (14)	O4—S2—C16—C21	−171.11 (12)
C3—C4—C5—C6	0.9 (2)	O3—S2—C16—C21	59.86 (13)
C9—O5—C6—C7	3.5 (2)	C1—S2—C16—C21	−53.66 (13)
C9—O5—C6—C5	−176.81 (16)	C21—C16—C17—C18	−1.1 (2)
C4—C5—C6—O5	177.80 (14)	S2—C16—C17—C18	172.98 (13)
C4—C5—C6—C7	−2.4 (2)	C16—C17—C18—C19	0.6 (3)
O5—C6—C7—C8	−178.93 (16)	C17—C18—C19—C20	0.3 (3)
C5—C6—C7—C8	1.3 (3)	C18—C19—C20—C21	−0.7 (2)
C6—C7—C8—C3	1.3 (3)	C19—C20—C21—C16	0.2 (2)
C4—C3—C8—C7	−2.8 (3)	C17—C16—C21—C20	0.8 (2)
C2—C3—C8—C7	176.83 (16)	S2—C16—C21—C20	−173.31 (12)

### Hydrogen-bond geometry (Å, °)

**Table:**

Cg is the centroid of the C16–C21 ring.

**Table:**

*D*—H···*A*	*D*—H	H···*A*	*D*···*A*	*D*—H···*A*
C8—H8···Cg1^i^	0.95	2.56	3.4835 (17)	164
C14—H14···O1^ii^	0.95	2.51	3.229 (2)	133
C21—H21···O3^i^	0.95	2.50	3.2695 (19)	138
C20—H20···O4^iii^	0.95	2.59	3.453 (2)	151

Symmetry codes: (i) −*x*, −*y*, −*z*+1; (ii) *x*+1, *y*, *z*; (iii) *x*−1, *y*, *z*.
